# Meddling with the Ears

**Published:** 1870-10

**Authors:** 


### MEDDLING WITH THE EARS.

Many persons are addicted to the very censurable habit of meddling with their ears, by picking at them with pins, pencils, tooth-picks and "ear-spoons" for the purpose of removing some imaginary cerumen, or with a view of relieving a slight itching sensation which always accompanies a dry condition of the external opening to the ear. The cerumen, or wax should never be removed from the ear, it is placed there for the purpose of lubricating the auditory passage and to prevent the introduction of particles of dust or insects. It is absolutely essential to the health of the parts, and should not be meddled with, and the picking and rubbing of the external opening to the ear, produces inflamation of the lining membrane, which results in an intolerable itching of the parts, and severe head-aches from the rubbing which is then indulged in.

Another very prevalent habit is that of syringing the ears. Much injury is done the hearing by means of this process, by having a stream of water thrown against the delicate drum of the ear, often rupturing it and causing such inflammation of its surface as to render it thickened or opaque, and so interfering with its proper vibration.

Others again, are forever cramming their ears with cotton, in order to prevent "taking cold." Particles of the cotton adhere to the cerumen and form a hard cement which soon excites irritability of the parts, hardens the remaining secretions of the ear, and obstructs the organ entirely.

We have frequently removed inpacted wax and cotton which had found a lodgment in the ear and had remained there without the knowledge of the wearer, for ten to fifty years, causing almost complete deafness. The most "astonishing cures of deafness are frequently performed by "traveling Eye and Ear Doctors," who simply remove such foreign substances—virtually "cleaning out the ears," for which kind and exceedingly "skillful" service they often receive handsome fees.

Never use oils in the ear "to soften the wax" or to cure deafness. Many persons pour all manner of trash into their ears for various purposes. Rattlesnake's oil, onion-juice, laudanum, tincture of camphor, and pepper are poured into the ear for the cure of as many different maladies. They never do good, but very often do much harm. To allay itching, several drops of pure glycerine may be placed in the ear and confined there with cotton, over night. To relieve ear-ache, equal parts of glycerine and laudanum, well shaken and warmed, by placing the bottle in hot water, may be confined in the ear in the same manner, but don't meddle with your ears in the manner above described.

